# Geospatial analysis of sports tourism resources in China's urban clusters: a case study of the Sichuan-Chongqing region utilizing GIS and the geographic detector

**DOI:** 10.3389/fspor.2024.1496469

**Published:** 2024-12-09

**Authors:** Xuefeng Tan, Zhiheng Liu, Lizhen Shi, Xiaoling Huang

**Affiliations:** ^1^ School of Physical Education, Southwest University, Chongqing, China; ^2^ School of Aerospace Science and Technology, Xidian University, Xi’an, China

**Keywords:** Sichuan-Chongqing urban agglomeration region, sports tourism resources, spatiotemporal distribution, urban planning, GeoDetector, Lorenz Curve

## Abstract

**Introduction:**

This study aims to elucidate the temporal and spatial distribution patterns of sports tourism resources within the Sichuan-Chongqing Urban Agglomeration, examining how these distributions reflect the developmental pathways of urban regions. The theoretical framework posits that the availability and allocation of sports tourism resources are influenced by various geographical and socio-economic factors, with topography, economic conditions, and population density playing pivotal roles in determining their distribution and development.

**Methods:**

Utilizing GeoDetector technology alongside indices such as the Geographic Concentration Index and the Disparities Index, we conducted a comprehensive analysis of the spatiotemporal dynamics of sports tourism resources, revealing that the distribution of these resources serves as a key indicator of urban development speed.

**Results:**

Our findings indicate that topographical features significantly influence sports tourism resource development differently in Chengdu compared to Chongqing. Furthermore, we identified a strong positive correlation between population density and the distribution of sports tourism resources, highlighting the motivational role of populated areas and emphasizing potential inequalities if attention is focused solely on densely populated regions.

**Discussion:**

This research provides novel insights into the complex relationships governing sports tourism resource distribution by employing advanced geo-analytical tools. It offers a strategic roadmap for equitable and sustainable urban development by addressing disparities in resource allocation, ultimately contributing to informed policy-making and enhanced regional resilience. This research aids in the strategic planning and sustainable development of sports tourism, providing a blueprint for urban development in the region that balances the needs of diverse populations and landscapes.

## 1 Introduction

Sports tourism, an activity where individuals engage in or spectate sports as the main purpose or content, has become a tourism phenomenon with extensive social, economic, cultural, and ecological impacts (1). Scholars have conducted extensive research around sports tourism (2). For instance, Yu et al. (3) utilized techniques such as the Gini coefficient and nearest neighbor index, based on data fusion and mining, to analyze the external environment of sports tourism development, the distribution of sports tourism resources, and the spatial structure of sports tourism, delving into the state of sports tourism spatial distribution. He and Wu (4), examined the sports tourism resources of the Grand Canal landscape environmental cultural belt from the perspective of ecological spatial development patterns, demonstrating that sports tourism supply must be market-oriented, integrating sports activities with tourism activities organically to develop various sports projects that meet tourists’ demands, achieving ecological benefits, economic benefits, and social benefits of ecological sports tourism. Li and Lyu (5), conducted a classification evaluation of ethnic traditional sports tourism resources based on machine learning, proposing that existing classifications of sports tourism resources are mostly artificially constructed based on the national standard tourism resource classification system. Traditional manual classification is inefficient and inaccurate, failing to reflect the characteristics of regional ethnic traditional sports tourism, and applied a Backpropagation (BP) model for sports tourism resource classification, achieving satisfactory results. Liu et al. (6) investigated the universal symbiosis and spatial hierarchy of sports tourism resources through Nearest Neighbor Index, Concentration Index, and Kernel Density Estimation, and found that the spatial analysis perspective greatly enhanced the comprehensive strength of the sports tourism industry and realized the sustainable economic sustainable development. Zuo et al. (7) utilized these two methods to explore spatial distribution patterns in their study of the spatial distribution patterns and influencing factors of sports tourism resources in China. Zhong et al. (8) conducted a comprehensive analysis of the spatial evolution characteristics of national sports industry bases within Chinese urban agglomerations, employing geo-detectors and additional methodologies to dissect the underlying influencing mechanisms. The study's findings underscore the significance of the economic base, market potential, and policy environment in their interactive role with other determinants, collectively enhancing the development and empowerment of sports resources. This research provides valuable insights into the multifaceted factors that contribute to the spatial dynamics of sports industry bases and highlights the interplay between economic, market, and policy factors in shaping the sports tourism landscape.

The Sichuan-Chongqing Region, located in the southwestern part of China, possesses strong development potential. Existing research has shown that the Chengdu-Chongqing economic circle is an important growth pole and new source of momentum for China's high-quality development (9, 10). With its unique geographical advantages and profound historical heritage, the Sichuan-Chongqing Region provides opportunities for the development of the tourism industry and has attracted academic attention regarding the development of its sports tourism industry. Zhang (11) studied the development of sports tourism in the Chengdu-Chongqing economic circle from the perspective of ethnic culture. Tian et al. (12), analyzed the development path of sports tourism in the Chengdu-Chongqing economic circle from the dual circulation perspective of international and domestic. These studies point out the development path of sports tourism in the Chengdu-Chongqing economic circle from a macro perspective, which is of research significance. However, they are insufficient to intuitively express the structure and distribution of sports tourism resources, spatial structure, and development potential of the region. Currently, there are studies on the tourism industry in the Sichuan-Chongqing Region that have intuitively and quantitatively analyzed resource distribution, etc. For example (13), studied the evolution of the spatial pattern of tourism flow and influencing factors in the Chengdu-Chongqing economic circle through spatial analysis, finding a significant “dual-core” bipolar differentiation effect in the tourism flow of the Chengdu-Chongqing economic circle, which was also confirmed in another study on spatiotemporal distribution characteristics (14). Feng (15) focused on the coupling of transportation accessibility and regional tourism economy in the Chengdu-Chongqing economic circle, finding that the coupling coordination level of tourism accessibility and tourism economic connection in the Chengdu-Chongqing economic circle is basically coordinated. In summary, the Sichuan-Chongqing Region has abundant sports tourism resources, and there are already studies on it, but the research is limited to the macro level and lacks intuitive and quantitative analysis, making it difficult to deeply explore the potential brought by its resource distribution, classification, and spatial structure, and to accurately analyze the factors hindering the development of the region.

In conclusion, the spatiotemporal distribution characteristics of sports tourism resources in the Sichuan-Chongqing Region and the analysis of influencing factors based on these characteristics are the goals of this study. Therefore, to address the issue of spatiotemporal distribution characteristics, we will use spatial analysis methods to deeply explore the following content:

(1) The temporal distribution characteristics of sports tourism resources in the Sichuan-Chongqing Region;
(2) The spatial distribution characteristics of sports tourism resources in the Sichuan-Chongqing Region;
(3) The analysis of influencing factors of sports tourism in the Sichuan-Chongqing Region.

## 2 Methods and materials

### 2.1 Methods

The Excel software is utilized for the quantitative organization of sports tourism resources in the Sichuan-Chongqing region. Spatial analysis methods within ArcGIS 10.8 software are employed to create maps illustrating the current geographical distribution of sports tourism resources in the region. Google Earth software is used for precise positioning of sports tourism resources in the Sichuan-Chongqing area to obtain accurate latitude and longitude coordinates. These coordinates are then imported into ArcMap software as XY point features. Spatial statistics such as the mean nearest neighbor index, geographic concentration index, disparity index, and kernel density estimation methods are applied to calculate density values and to conduct research on influencing factors.

#### 2.1.1 Mean nearest neighbor index

The Mean Nearest Neighbor Index is a metric used to describe the degree of spatial clustering or dispersion of point-like phenomena, comparing the pattern of aggregation of a particular object within a region to that of a random distribution (16). The ANNI is an indispensable metric in spatial analysis that quantifies the degree of pattern clustering or dispersion within a dataset, thereby revealing the irreplaceability of specific locations. The unique value of ANNI lies in its ability to express both the quantity and length of flows arriving at a location with a single indicator, effectively revealing the multi-dimensional characteristics of flow data. The formula as follows:

(1) $$ANNI=\frac{ANNO}{ANNE}=\frac{\sum_{i=1}^{n}\min(\mathrm{diy})}{n}/\frac{0.5}{\sqrt{n/A}}=\frac{2\sum_{i=1}^{n}\min(\mathrm{diy})}{\sqrt{n/A}}$$

where the ANNO represents the average observed distance, ANNE denotes the expected average distance under random distribution conditions, min(d) is the distance between any sports tourism resource and its nearest neighboring sports tourism resource, *n* is the total number of sports tourism resources, and A is the total area of the study region. An ANNI value less than 1 indicates that the spatial distribution pattern of the objects is clustered; the smaller the value, the greater the degree of clustering. Conversely, an ANNI value greater than 1 suggests that the objects tend to be dispersed in space.

#### 2.1.2 Geographic concentration index

The Geographic Concentration Index is a pivotal metric for assessing the degree of concentration of the study subject (17). It can reveal the irreplaceability of specific locations, reflect information on population activities, and is of significant importance for understanding the unique value of places and conducting people-oriented planning. The formula for this index is as follows:

(2) $$G=100\times \sqrt{\sum_{i=1}^{n}{\left(\frac{Xi}{T}\right)}^{2}}$$

Where the G denotes the Geographic Concentration Index for sports tourism resources in the Sichuan-Chongqing region, where Xi is the count of sports tourism resources in the i-th region, T is the total number of sports tourism resources, and *n* is the total number of regions under study. The index G ranges from 0 to 100, with higher values indicating a more concentrated distribution of sports tourism resources, and lower values indicating a more dispersed distribution.

#### 2.1.3 Inequality Index

The Inequality Index is a measure that reflects the degree of evenness in the distribution of the study subject across different regions (18). It possesses necessity and irreplaceability in revealing regional development disparities and uneven resource allocation, offering a scientific basis for policy formulation that fosters social equity and coordinated regional development. The formula for this index is as follows:

(3) $$S=\frac{\sum_{i=1}^{n}\Upsilon i-50(n+1)}{100n-50(n+1)}$$

where the *n* represents the total number of regions, and Yi denotes the percentage of sports tourism resources in the i-th region of the Sichuan-Chongqing area relative to the total count, with the regions ordered from the highest to the lowest percentage. The cumulative weight of the i-th region after this ordering is considered. The Inequality Index, denoted as S ranges from 0 to 1. A value of S closer to 1 indicates a higher concentration of all sports tourism resources in a single region, while a smaller S value suggests a more even distribution across the regions.

#### 2.1.4 Kernel density estimation

Kernel Density Estimation (KDE) is a non-parametric method for analyzing the density of geographical elements within their surrounding areas. This method assigns different weights to the sample points within the study area through a kernel function, thereby producing a smoother density map that reveals the density attributes of the study area. The formula for KDE is as follows (19):

(4) $$F(x)=\left(\frac{1}{nh}\right)\sum_{i=1}^{n}k[(x\text{-}\mathrm{xi})/h]$$

where the ℎ represents the bandwidth (where h > 0), *n* is the total number of sports tourism resources within the Chengdu-Chongqing dual-city economic circle, and (X−Xi) denotes the distance from the point data X to the estimation point Xi. The larger the value of F(x), the more concentrated the distribution of sports tourism resources is indicated.

#### 2.1.5 GeoDetector

The GeoDetector is a method for detecting spatial heterogeneity, serving to analyze the influencing factors of a certain phenomenon as well as the interactions among multiple factors (20). Geographic GeoDetector is an advanced spatial statistical analysis tool that quantifies the spatial correlations among geographical elements and detects the underlying factors and their interactions driving geographical phenomena. Compared to traditional methods, the Geographic Detector offers superior flexibility and applicability, capable of handling non-linear relationships without stringent statistical assumptions, thereby providing a more direct and effective approach for the analysis of geospatial data. The formula for the GeoDetector is as follows:

(5) $$q=1-\frac{\sum_{h-1}^{L}N_{h}Q_{\;h}^{2}}{NQ^{2}}=1-\frac{SST}{SST}$$

where ℎ=1,..,L represents the stratification of the dependent variable Y or the independent variable X; Q2 h and Q2 are the variances of Y within the stratum and for the entire region, respectively. SSW and SST denote the sum of within-stratum variances and the total variance for the entire region, respectively. The value of q ranges from 0 to 1, where a larger q indicates a stronger explanatory power of X on the spatial differentiation of Y. In extreme cases, a q value of 1 suggests that the spatial distribution of Y is entirely controlled by X, while a q value of 0 indicates no relationship between them.

### 2.2 Materials

#### 2.2.1 Data sources and processing

The research data is sourced from the official websites of the Chengdu and Chongqing municipal governments, which disclose sports tourism resources. These resources encompass sports tourism demonstration bases, high-quality sports events, and featured sports tourism routes. The official websites are updated annually to showcase the high-quality projects of the year. Regardless of whether they appear continuously, their essence as sports tourism resources is recognized, and they are therefore included in the final spatial analysis database.

The study area includes the administrative divisions of Sichuan Province and Chongqing Municipality, which collectively govern 115 county-level administrative units, covering a total area of 568,452 km^2^. This area constitutes an important economic and cultural region in Southwest China. During the statistical process, the distribution of these resources within the Sichuan-Chongqing region is counted on an individual basis to provide a data foundation for in-depth discussion and analysis in this study.

## 3 Results

### 3.1 Characteristics of the temporal distribution of sports tourism resources in the Sichuan-Chongqing region

The temporal segmentation of sports tourism is an essential means to understand the development history, formation mechanisms, and governmental decision-making directions. It is also a prerequisite for promoting the high-quality development of sports tourism and aligning with the high-quality development of the sports industry. The high-quality development of the Sichuan-Chongqing region has been marked by the promulgation of the “Chengdu-Chongqing Economic Circle Development Plan Outline” in 2021 (21), and the development of the sports tourism industry follows suit (22). Therefore, this study uses policy documents as milestones and classifies the development of sports tourism into two stages: the exploratory development stage and the high-quality development stage, based on type and quantity.

#### 3.1.1 Exploratory and upward development stage

Before the promulgation of the “Chengdu-Chongqing Economic Circle Development Plan Outline,” the development of sports tourism in the Sichuan-Chongqing region has shown a significant upward trend and unique regional characteristics (23). Its temporal distribution characteristics are particularly prominent, manifesting as distinct seasonality and periodicity. The formation of this trend is closely related to the natural geographical conditions of the region and also benefits from the deep integration of policy orientation and cultural characteristics.

Firstly, the region's unique natural landscapes provide a natural advantage for the development of sports tourism. In spring and autumn, the pleasant climate is the best time for outdoor activities, attracting a large number of enthusiasts to activities such as hiking, climbing, and cycling. In summer, the Sichuan-Chongqing region becomes a summer retreat, with water sports and high-altitude outdoor activities being particularly popular. In winter, the snow resources in some areas provide conditions for skiing and snow festivals, attracting a large number of tourists (24). This seasonal characteristic not only reflects the natural laws of sports tourism activities but also demonstrates the rational use of natural resources in the Sichuan-Chongqing region. Secondly, the development of sports tourism in the region also shows a certain periodicity (25). Various regularly held sports events, such as the Chengdu International Marathon and the Chongqing International Climbing Competition, as well as traditional festival sports activities like the Dragon Boat Race during the Dragon Boat Festival, have formed a stable periodic pattern that attracts tourists. These activities not only enrich the content of sports tourism but also enhance its cultural connotation and attractiveness. In terms of policy orientation, the governments of the Sichuan-Chongqing region actively respond to the national call for the development strategy of sports tourism, promoting the development of the sports tourism industry through policy guidance and financial support. The timing and phased goals in policy implementation are in line with the seasonality and periodicity of sports tourism, further strengthening its temporal distribution characteristics. In addition, the government has also promoted the integrated development of sports tourism with other industries, such as the combination of sports tourism with eco-tourism and cultural tourism, injecting new vitality into the development of sports tourism. The region's rich cultural heritage and traditional sports projects, such as martial arts, dragon boat races, and ethnic dances, have been transformed and innovated through modernization, forming sports tourism products with regional characteristics. These products not only provide tourists with the opportunity to deeply understand and experience the local culture but also further enrich the content and form of sports tourism.

However, the development of sports tourism in the Sichuan-Chongqing region also faces some challenges, such as insufficient coordination in the use of sports tourism resources in the region, poor service quality, and lack of refined projects. These challenges have to some extent restricted the further development of sports tourism but also indicate great potential for development and opportunities.

In summary, before the promulgation of the “Chengdu-Chongqing Economic Circle Development Plan Outline,” the development of sports tourism in the Sichuan-Chongqing region showed dominant characteristics of seasonality and periodicity in its temporal distribution, while also showing significant regional characteristics and an upward trend.

#### 3.1.2 High-quality development stage (since 2021)

**Figure 1:**
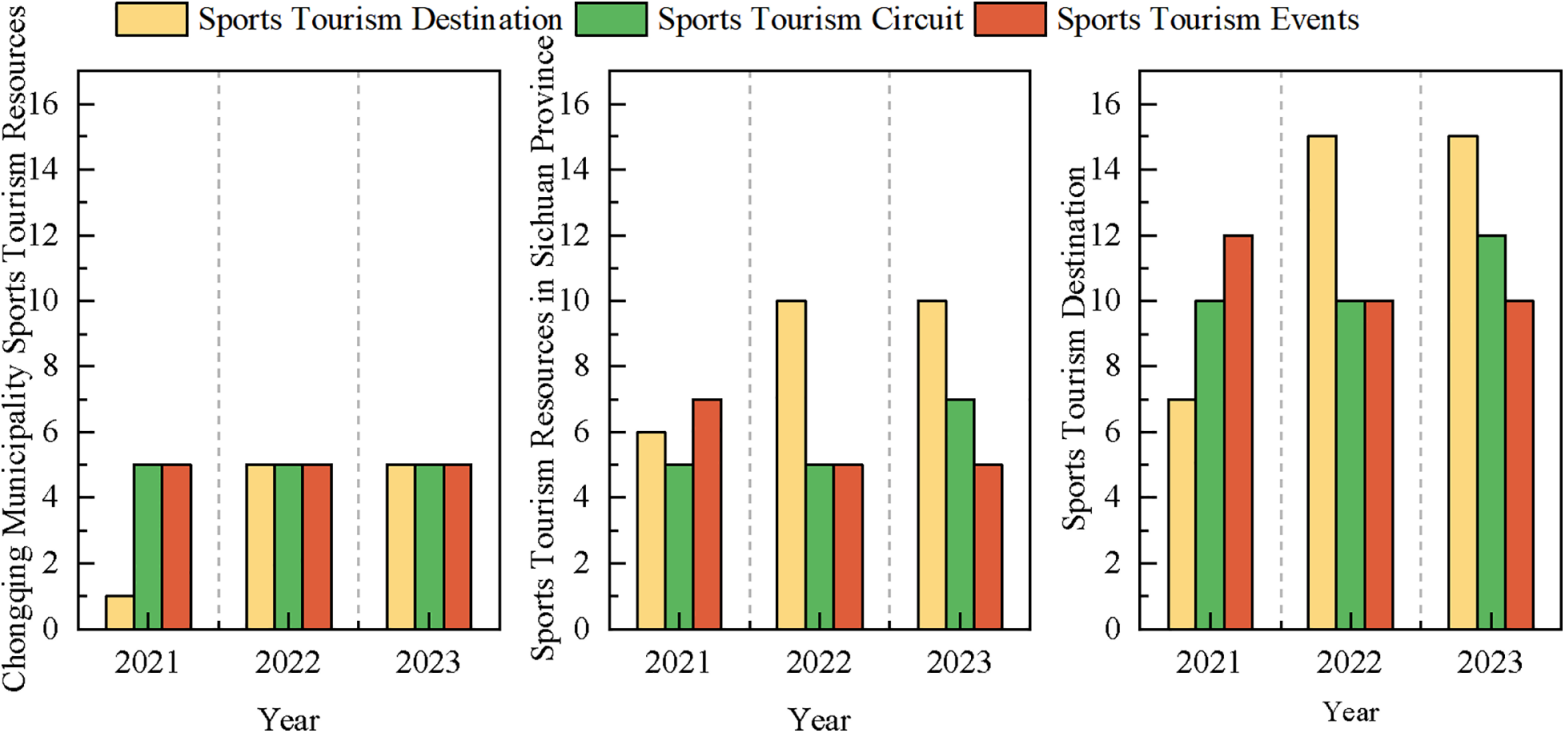
Trends in sports tourism resources in the Chengdu-Chongqing region from 2021 to 2023.

The number of sports tourism bases, routes, and events promoted by the Sichuan-Chongqing region increases or is continuously updated every year, and this study counts the number of promotions each year. As shown in Figure 1, since the promulgation of the “Chengdu-Chongqing Economic Circle Development Plan Outline,” the number of sports tourism demonstration bases in Chongqing has increased significantly from 2021 to 2022, while sports tourism events and routes have remained stable. In Sichuan Province, the number of sports tourism demonstration bases increased significantly from 2021 to 2022, and remained stable by 2023; sports tourism routes remained stable from 2021 to 2022, with a slight increase in 2023, and sports events experienced a slight decline from 2021 to 2022, then remained stable. Chongqing has a smaller administrative area than Sichuan Province, and therefore has fewer resources in terms of volume. The most significant change is in the sports tourism demonstration bases, which may be due to China's strong promotion of the construction of tourism demonstration bases. Overall, sports tourism bases, routes, and events in the Sichuan-Chongqing region have been steadily developing over the past three years, and the reasons for this development are roughly as follows:

The emergence of regional synergy effects, thanks to the regional integration development strategy proposed in the “Outline Plan” (26). The governments of Sichuan and Chongqing have been increasingly cooperating in the field of sports tourism, achieving diversification and regional characteristics of sports tourism products through resource sharing, collaborative planning, and joint marketing. For example, jointly hosting cross-regional sports events not only enhances the scale and influence of the events but also promotes regional linkage of sports tourism, attracting more domestic and foreign tourists.

The transformation to high-quality development (27) is reflected in the continuous improvement of the quality of sports tourism products and services. Relying on its unique natural landscapes and cultural resources, the Sichuan-Chongqing region has developed a series of high-quality sports tourism projects, such as off-road racing, water sports, and ice and snow tourism. These projects not only meet consumers’ diverse needs for health, leisure, and adventure but also promote the development of sports tourism services towards specialization and personalization.

The Sichuan-Chongqing region has also made significant progress in infrastructure construction, including that of sports tourism. By increasing investment, improving transportation networks (28), enhancing service facilities, and strengthening the level of informatization, a more convenient and comfortable tourism experience has been provided for tourists.

### 3.2 Analysis of the distribution characteristics of sports tourism in the Sichuan-Chongqing region

**Figure 2:**
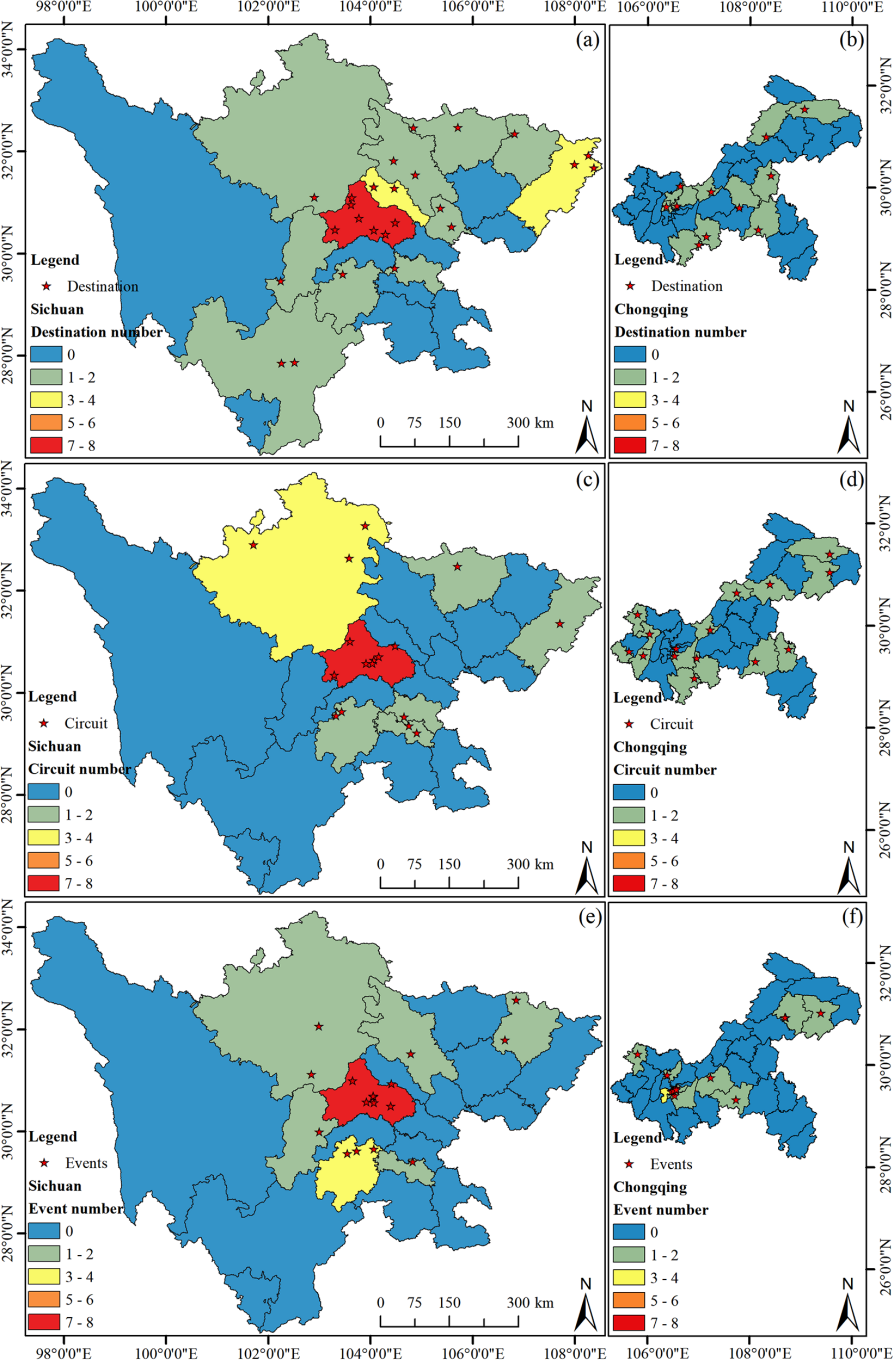
Distribution of the sports destination, circuit, and events in Sichuan-Chongqing area. **(a)** The distribution of the destination in Sichuan, **(b)** the distribution of the destination in Chongqing, **(c)** the distribution of the circuit in Sichuan, **(d)** the distribution of the circuit in Chongqing, **(e)** the distribution of the events in Sichuan, **(f)** the distribution of the events in Chongqing.

To accurately present the spatial distribution of sports tourism resources in the Sichuan-Chongqing region, geographical distribution maps at the provincial and municipal levels of Sichuan Province and Chongqing City have been created (see Figure 2), highlighting the intuitive spatial distribution of sports tourism resources. Additionally, according to th Equations 1–4, the Inequality Index, Geographic Concentration Index, and Mean Nearest Neighbor Index (see Table 1) are employed to test the degree of equilibrium and distribution types of sports tourism resources, providing data support for the study of the overall distribution characteristics of geographical elements.

**Table 1:** The equilibrium index of sports tourism resources, geographical concentration index, average nearest neighbor index, and average value.

Name	Region	ANNI	G	S	F(x)
Destination	Sichuan	0.67	36.08	0.59	0.54
	Chongqiong	1.20	30.16	0.73	1.19
Circuit	Sichuan	0.74	48.86	0.76	0.35
	Chongqiong	1.23	25.81	0.62	1.57
Events	Sichuan	0.57	48.86	0.76	0.36
	Chongqiong	0.59	33.33	0.79	1.88

#### 3.2.1 Distribution characteristics of sports tourism demonstration bases in the Sichuan-Chongqing region

Figure 2a illustrates the spatial distribution of sports tourism demonstration bases in Sichuan Province. The base points are predominantly located in the central and eastern parts of the province, with no demonstration bases in the western region, which correlates with the higher elevation of the terrain there. Further analysis reveals that as the provincial capital, Chengdu City has the highest number of sports tourism demonstration bases (a total of 7), followed by Dazhou City (3), and Guangyuan City, Suining City, Deyang City, and Liangshan Prefecture each have two, with the remaining areas having one each. As shown in Figure 2b, compared to Sichuan Province, Chongqing City, despite its smaller area, has a similar number of sports tourism demonstration bases. The reason for analyzing Chongqing City separately is that it is geographically adjacent to Sichuan, and as a directly-administered municipality, it better reflects the characteristics of the regional sports tourism industry. The distribution of sports tourism bases in Chongqing generally shows a trend of higher concentration in the central areas and fewer in the peripheral areas, without any particular concentration in one specific area. Wuxi County, Kai County, Shizhu County, Pengshui County, Nanchong City, Wansheng District, Yuzhong District, Jiangbei District, and Hechuan City each have one base.

#### 3.2.2 Distribution characteristics of high-quality sports tourism routes in the Sichuan-Chongqing region

As shown in Figure 2c, the color intensity indicates that high-quality sports tourism routes in Sichuan Province are mainly distributed in the central and northern parts, with no high-quality routes in the western and southern parts. Chengdu City has the highest number, with a total of 7 routes. This number corresponds with the quantity of sports tourism demonstration bases, indicating a certain degree of correlation. Aba Prefecture has 3 routes, Leshan City and Zigong City each have two, and Guangyuan City, Neijiang City, and Dazhou City each have one. As depicted in Figure 2d, the distribution of high-quality sports tourism routes in Chongqing City is characterized by fewer in the center and more around the edges, with a relatively even distribution and no trend of concentration. Wuxi County, Fengjie County, Qianjiang County, Pengshui Autonomous County, the main urban area, Caijiang County, Rongchang County, Yongchuan City, Tongliang County, and Tongnan County each have one route, which is relatively consistent with the distribution of sports tourism demonstration bases.

#### 3.2.3 Distribution characteristics of sports events in the Sichuan-Chongqing region

As shown in Figure 2e, the locations for sports events in Sichuan Province are relatively concentrated, mainly in Chengdu City. The selection of event locations that suit the terrain is crucial and varies with the type of event. Most events concentrated in Chengdu City are those that do not depend on the terrain. Leshan City has 3 events, Aba Prefecture and Bazhong City have 2 events, and Zigong City, Ya'an City, and Mianyang City each have 1 event. These events are mostly terrain-dependent, such as the Emei Mountain Cross-Country Challenge. As shown in Figure 2f, sports events in Chongqing City are concentrated in the main urban area, with very few in other districts and counties, most of which have none.

### 3.3 Analysis of the distribution characteristics of sports tourism in the Sichuan-Chongqing region

As shown in Table 1, regarding the Geographic Concentration Index, the distribution of sports tourism resources in Sichuan Province is less concentrated than that in Chongqing City, indicating a higher degree of dispersion, which may be related to the administrative areas of the two regions. Specifically, (1) the indices for sports tourism resources in Sichuan Province are relatively close, at 36.80, 48.86, and 48.86, respectively. In contrast, Chongqing exhibits indices of 30.16, 25.81, and 33.33, suggesting a more uniform distribution of sports tourism demonstration bases, routes, and events; (2) From the perspective of the Inequality Index, both Chongqing and Sichuan show an increasing trend of inequality for demonstration bases, routes, and events, with Sichuan's indices at 0.59, 0.76, and 0.76, respectively. Chongqing's indices for sports tourism demonstration bases, routes, and events reach 0.73, 0.62, and 0.79, respectively, showing a highly uneven distribution compared to Sichuan Province; (3) Regarding the Mean Nearest Neighbor Index, the ANNI values for sports tourism demonstration bases, routes, and events in Sichuan Province are 0.67, 0.74, and 0.57 (ANNI < 1), respectively, indicating an aggregated distribution pattern, and both are statistically significant (*P* < 0.01). Thus, the distribution of sports tourism resources in Sichuan Province is significantly aggregated. The ANNI values for Chongqing's sports tourism demonstration bases and routes are 1.20 and 1.23 (ANNI >1), respectively, indicating a dispersed distribution, while the ANNI for events is 0.59 (ANNI <1), showing an aggregated distribution.

In summary, the distribution of sports tourism resources in the Sichuan-Chongqing region is related to the spatial scale. Except for events, the difference in the Inequality Index is greater than that of the Geographic Concentration Index in the region. Sichuan Province's Mean Nearest Neighbor Index consistently shows a significant aggregated pattern, while Chongqing's shows a dispersed pattern. Apart from events, the overall spatial distribution of Chongqing's sports tourism presents characteristics of high aggregation and low degree of equitability, which is consistent with the distribution map (Figure 2).

The Lorenz Curve is a statistical tool used to characterize and measure the degree of equality in resource distribution, proposed by the American statistician Max Otto Lorenz in 1905. The curve illustrates the inequality of distribution by arranging individuals or households in ascending order of income or wealth, and then plotting the cumulative percentage of the population against the cumulative percentage of income or wealth. In an ideal case of equal distribution, the Lorenz Curve would be a straight line of proportionality, also known as the “line of equality.” However, the actual Lorenz Curve always lies below the line of equality, and the greater the area between the curve and the line of equality, the greater the degree of inequality in distribution.

**Figure 3:**
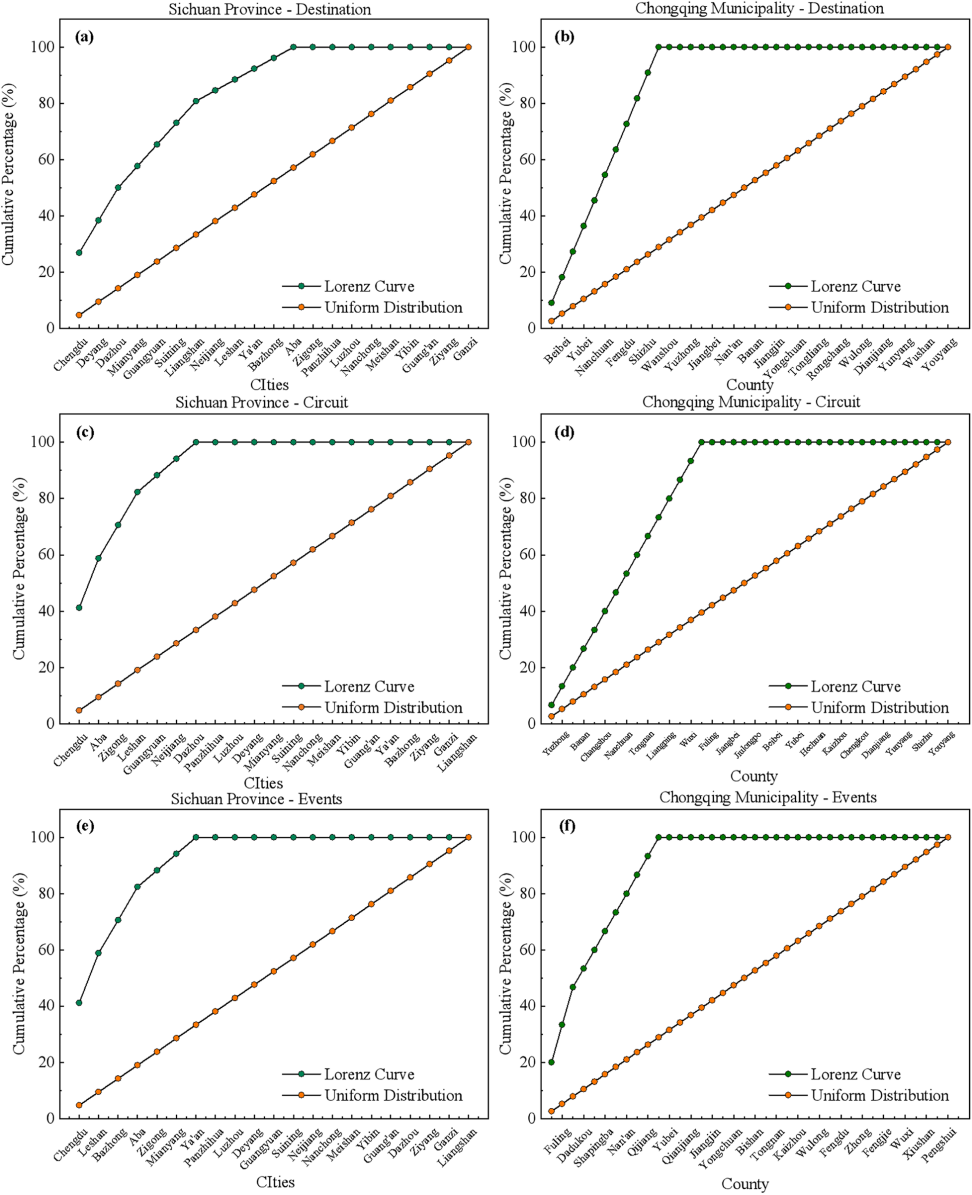
Lorenz curve analysis of sports tourism resources in Sichuan and Chongqing. **(a)** the Lorenz curve distribution of destination in Sichuan, **(b)** the Lorenz curve distribution of destination in Chongqing, **(c)** the Lorenz curve distribution of the circuit in Sichuan, **(d)** the Lorenz curve distribution of the circuit in Chongqing, **(e)** the Lorenz curve distribution of the events in Sichuan, **(f)** the Lorenz curve distribution of the events in Chongqing. Lorenz curve distribution of sports tourism in the Sichuan-Chongqing region. Aba, Ganzi, Liangshan in the Figure 3. **(a,c,e)**, are Aba Tibetan and Qiang Autonomous Prefecture, Ganzi Tibetan Autonomous Prefecture, Liangshan Yi Autonomous Prefecture, respectively.

To compare the differences in the Lorenz Curves of sports tourism between the Chengdu and Chongqing regions, the traditional village Lorenz Curves were generated based on statistical data (Figure 3). The Lorenz Curves are relatively far from the line of uniform distribution, with a significant curvature, indicating an uneven distribution of sports resources across various regions within the province. As can be seen from the figure, in Sichuan Province, the distribution of sports tourism demonstration bases is concentrated in Chengdu, Deyang, and Dazhou; for sports tourism routes, Chengdu, Aba Prefecture, and Zigong have a larger share; and for sports events, Chengdu, Leshan, and Bazhong have a higher concentration. Within Chongqing, the distribution of sports tourism demonstration bases is denser in Beibei District, Yubei District, and Nanchuan District; for sports tourism routes, the distribution is more abundant in Yuzhong District, Banan District, and Changshou District; and for sports events, Fuling District, Dadukou District, and Shapingba District have a higher proportion.

#### 3.3.1 Kernel density analysis of sports tourism resources in the Sichuan-Chongqing region

**Figure 4:**
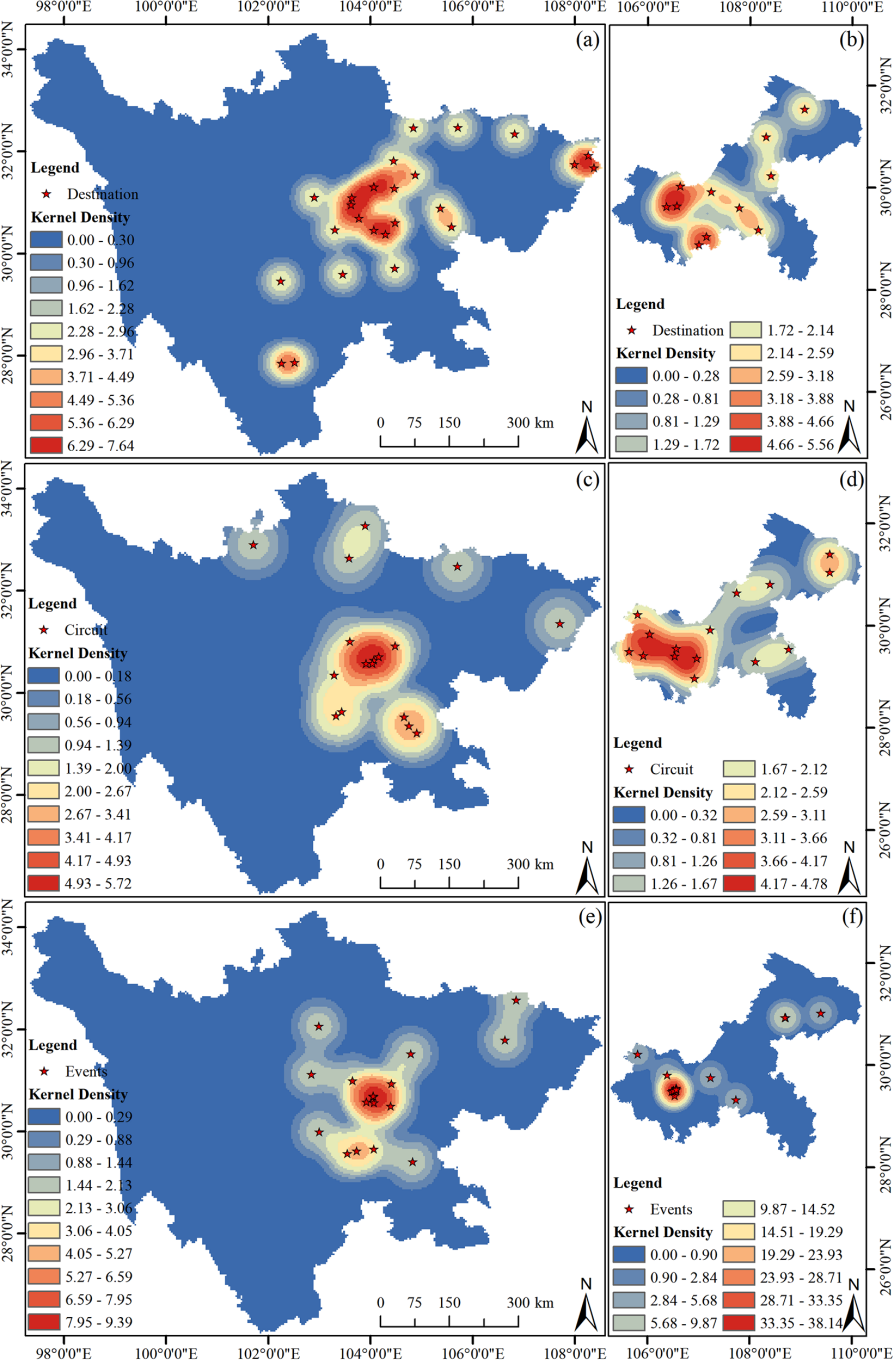
Kernel density distribution of sports tourism resources in Sichuan and Chongqing regions. **(a)** The distribution of the destination in Sichuan, **(b)** the distribution of the destination in Chongqing, **(c)** the distribution of the circuit in Sichuan, **(d)** the distribution of the circuit in Chongqing, **(e)** the distribution of the events in Sichuan, **(f)** the distribution of the events in Chongqing.

The distribution of sports resources in Sichuan Province is generally concentrated, exhibiting a spatial pattern of aggregation in the central and eastern parts and scarcity in the western regions. To further illustrate the spatial agglomeration characteristics of the national sports industry base, the ArcToolbox in ArcGIS was utilized, selecting an appropriate bandwidth and cell size to obtain the kernel density maps of the spatial distribution of demonstration bases and units (Figure 4).

According to Figures 4a,c,e, the sports tourism demonstration bases, routes, and events in Sichuan Province show two major agglomeration areas on the kernel density map, centered around Chengdu City, with the highest kernel density values. The central areas reach 6.29–7.64, 4.93–5.72, and 7.95–9.39, forming high-density regions; the area bordering Jing-Ji (Beijing-Hebei) has a secondary high-density area with kernel density values of 0.603–0.903. Overall, the kernel density map of sports tourism resources in Sichuan Province is predominantly characterized by high density, with few low-density areas. According to Figures 4b,d,f, apart from events, the distribution of sports tourism resources in Chongqing City is relatively uniform, showing a high-density area mainly in the central urban area, reaching 4.66–5.56 and 4.17–4.78, respectively. Apart from events, the kernel density distribution of sports tourism resources in Chongqing City generally exhibits a spatial pattern of “relatively uniform distribution with high-density areas in the central urban district.” The distribution of sports tourism events in Chongqing City is overly concentrated, showing a high-density area mainly in the central urban area, with values as high as 33.35 to 38.14.

#### 3.3.2 Buffer zone analysis of sports tourism resources in the Sichuan-Chongqing region

**Figure 5:**
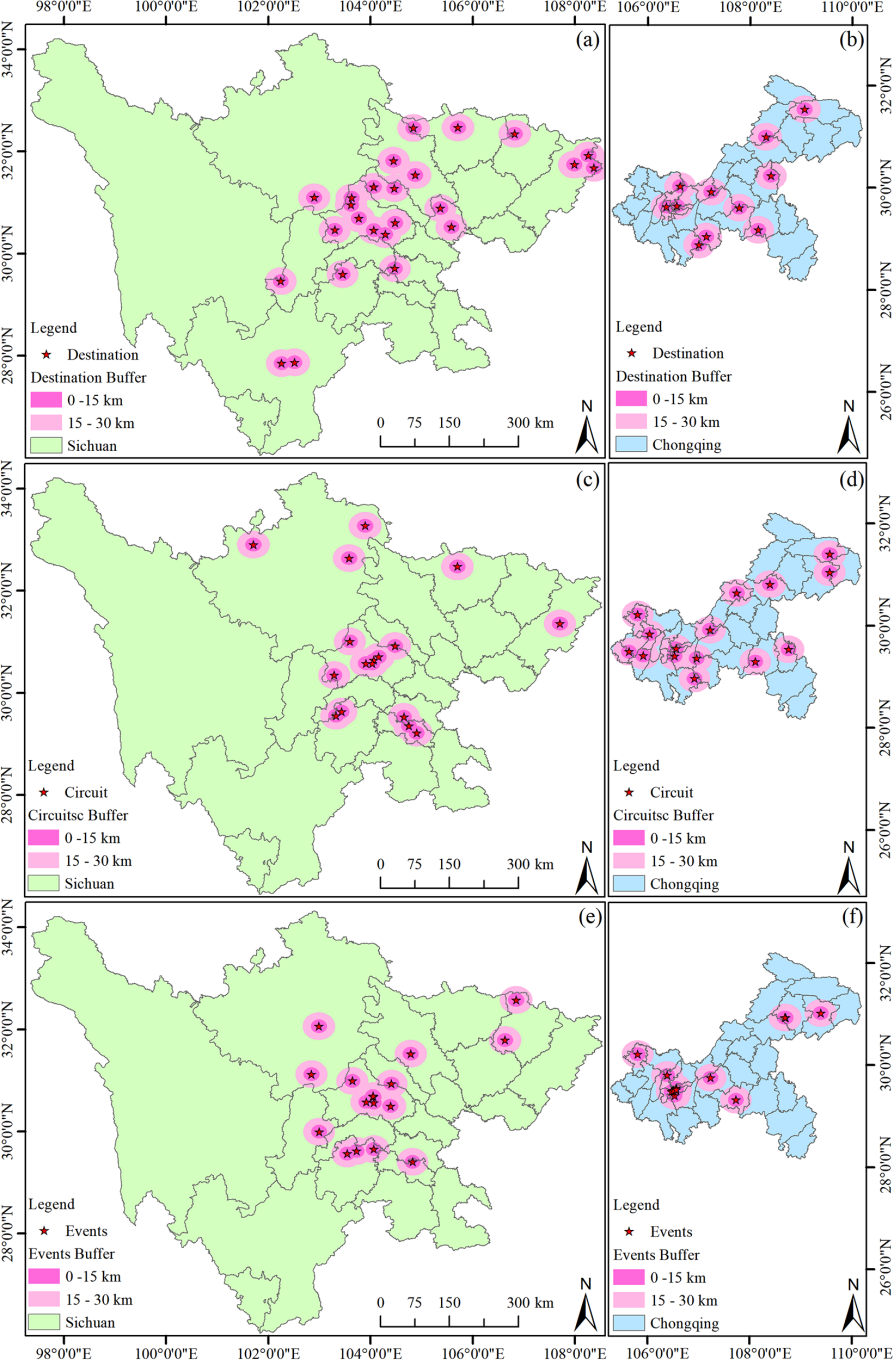
Kernel density distribution of sports tourism resources in Sichuan and Chongqing regions. **(a)** The distribution of the destination in Sichuan, **(b)** the distribution of the destination in Chongqing, **(c)** the distribution of the circuit in Sichuan, **(d)** the distribution of the circuit in Chongqing, **(e)** the distribution of the events in Sichuan, **(f)** the distribution of the events in Chongqing.

As depicted in Figure 5, this study categorizes and statistically analyzes the sports tourism resources in the Sichuan-Chongqing region, creating buffer zones with radii of 15 km and 30 km centered around each resource. It can be observed from the figure that the radiating areas of the sports tourism resources in Sichuan Province are relatively small compared to the entire administrative region of the province, indirectly indicating a concentrated distribution of sports tourism resources, which further corroborates the computational results presented in Table 1. In contrast, Chongqing exhibits a more uniform distribution with larger coverage areas, which may be related to the size of the administrative area, indirectly suggesting a more evenly distributed pattern of sports tourism resources in Chongqing.

#### 3.4.1 The impact of transportation conditions on sports tourism in the Sichuan-Chongqing region

**Figure 6:**
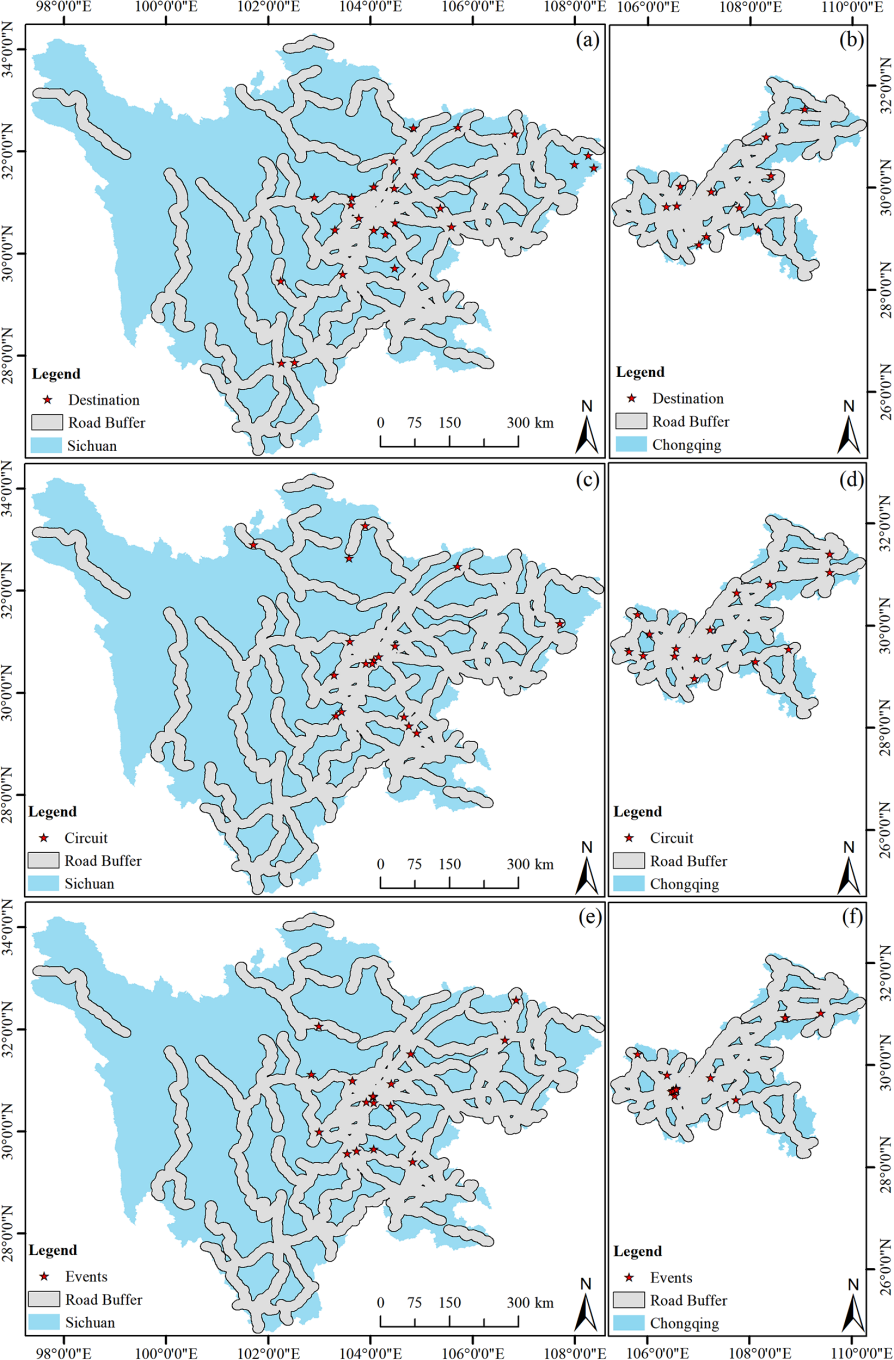
Buffer area of road with a radius of 10 km of sports tourism resources in Sichuan and Chongqing regions. **(a)** The buffer area of the destination in Sichuan, **(b)** the buffer area of the destination in Chongqing, **(c)** the buffer area of the circuit in Sichuan, **(d)** the buffer area of the circuit in Chongqing, **(e)** the buffer area of the events in Sichuan, **(f)** the buffer area of the events in Chongqing.

Transportation and communication serve as the foundation and vector for various interrelated activities and are a critical link for the collaborative interaction of labor, production resources, and technological elements (29). They are also an important factor that constrains the development of the tourism industry (30). Utilizing the buffer analysis tool in ArcGIS 10.8, with national highways as the axis and selecting a 15 km buffer radius, a national highway buffer zone layer was created and overlaid with the sports tourism resource point data map to generate a map of the quantity of sports tourism resources in relation to the main highway buffer zones in the Sichuan-Chongqing region (Figure 6). Overall, it can be observed that sports tourism resources in the Sichuan-Chongqing region are distributed in areas with dense transportation networks, and virtually every sports tourism resource is located near a national highway.

The transportation routes in Sichuan and Chongqing have influenced the distribution of sports tourism resources, mainly in three aspects: accessibility, environment, and temporal efficiency. Accessibility is a fundamental condition for the development of sports tourism, referring to the convenience with which travelers can reach sports tourism destinations (31). Sichuan and Chongqing have improved the accessibility of sports tourism destinations by continuously optimizing their transportation networks. For instance, the Ba Nan Cultural Landscape Sports Tourism Route in Chongqing has been developed into a high-quality sports tourism route, with planning and construction that fully consider the accessibility needs of visitors. Environmental factors also play a significant role in the distribution of sports tourism resources. In the planning and construction of transportation routes, Sichuan and Chongqing have focused on ecological protection and environmental integration, combining transportation development with the protection of the natural environment through green transportation and sustainable tourism practices. Such environmentally friendly transportation routes not only provide sports tourists with a harmonious coexistence with nature but also protect the ecological environment of the destination, ensuring the long-term sustainability of sports tourism resources. Temporal efficiency is another key indicator for measuring the performance of transportation networks. Sichuan and Chongqing have improved the operational efficiency of their transportation networks by optimizing traffic management and services, reducing travel time. Fast transportation connections not only provide visitors with more time to engage in sports activities and enjoy tourism experiences but also ensure rapid response in emergency situations, enhancing the safety and reliability of sports tourism (32). Additionally, the transportation networks in Sichuan and Chongqing have played a key role in regional collaborative development. Through the integration of regional transportation, they have promoted the sharing and complementarity of sports tourism resources and strengthened cooperation and linkage between regions.

#### 3.4.2 The impact of GDP distribution on sports tourism in the Sichuan-Chongqing region

The economic foundation is an inductive factor that affects the agglomeration of tourism, and robust economic strength is conducive to promoting the development of tourism (33). It is also one of the important factors that constrain the development of sports tourism. For instance, economic strength provides the necessary financial support for the construction of tourism infrastructure, including the improvement of transportation networks, accommodation facilities, and tourism service facilities (34), thereby enhancing the accessibility and attractiveness of tourist destinations. Secondly, economic prosperity offers ample financial security for tourism market promotion activities, enabling tourist destinations to enhance their visibility globally through advertising, public relations activities, and other means (35). Furthermore, economic investment also promotes the improvement of tourism service quality, increasing visitor satisfaction and loyalty through professional training and service innovation (36).

**Figure 7:**
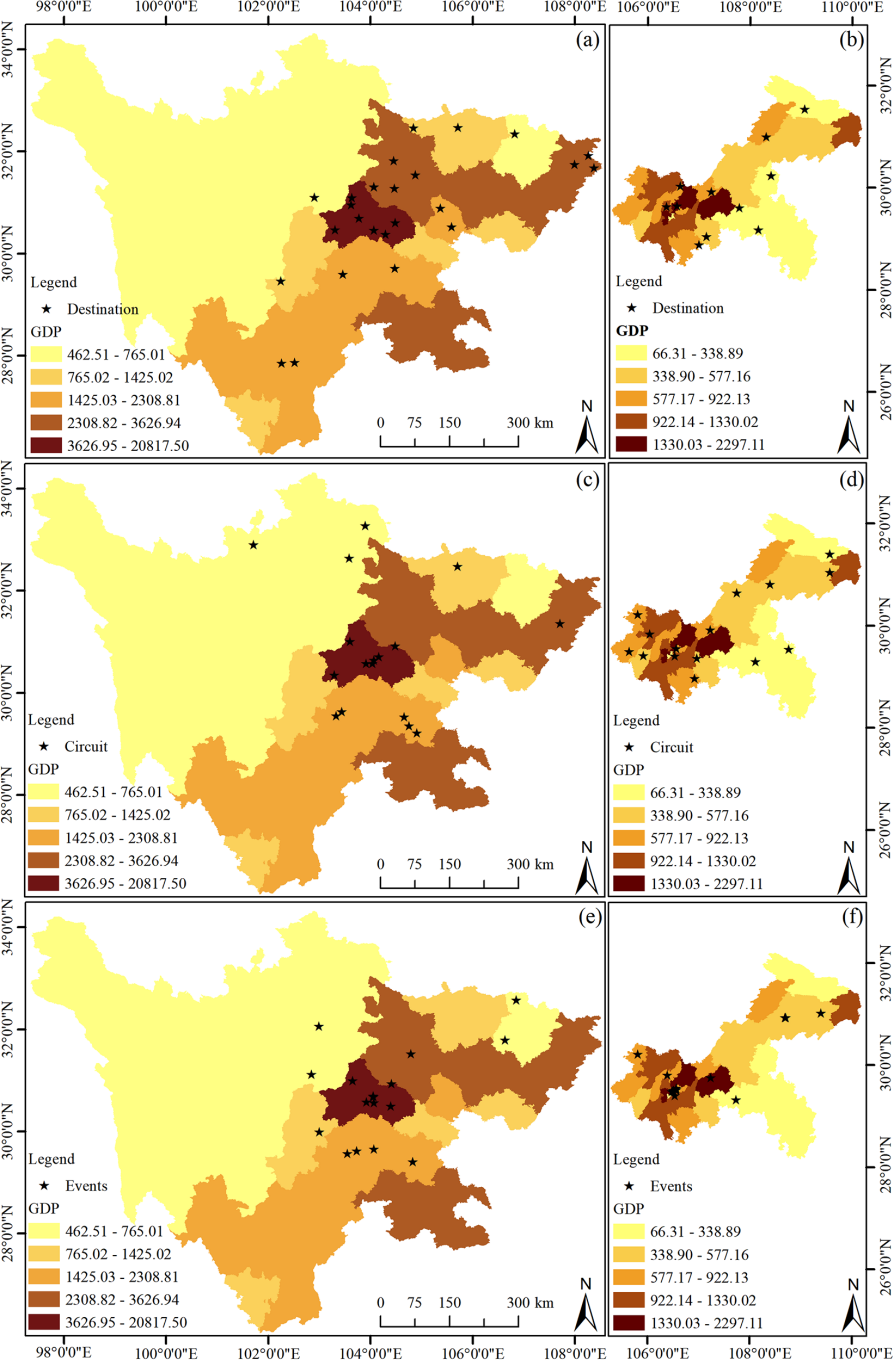
GDP effect on sports tourism resources in Sichuan and Chongqing regions. **(a)** the GDP effect on the destination in Sichuan, **(b)** the GDP effect on the destination in Chongqing, **(c)** the GDP effect on the circuit in Sichuan, **(d)** the GDP effect on the circuit in Chongqing, **(e)** the GDP effect on the events in Sichuan, **(f)** the GDP effect on the events in Chongqing.

To verify the relationship between sports tourism resources and regional GDP in the Sichuan-Chongqing area, the GDP of various districts and cities in Sichuan Province and Chongqing for the year 2022 was used to represent the development level of each region. ArcGIS 10.8 was employed to create a GDP map for each region, using the natural breaks classification method. This map was then overlaid with the point data map of demonstration bases and units to obtain a GDP level map of sports tourism resources in the Sichuan-Chongqing region (Figure 7).

As can be seen from Figure 7, there is a certain correlation between the spatial distribution of sports tourism resources and regional GDP in Sichuan, where darker colors represent a higher concentration of sports tourism resources. Sports tourism resources in Sichuan Province are mainly clustered in Chengdu City and radiate outward from there. In contrast, the distribution of sports resources in Chongqing is more dispersed, and no significant correlation is observed in the figure. Therefore, the Spearman correlation analysis method was further employed to analyze the relationship between the two. The results are shown in Table 2, indicating that there is a correlation between GDP and the distribution of sports tourism resources in Sichuan, while no correlation exists in Chongqing. This may be related to the terrain of Chongqing itself, which is rich in natural resources. However, areas with significant sports resources such as high mountains and large rivers have relatively lagging urbanization and commercial development, thus forming the current situation.

**Table 2:** An analysis of the correlation between GDP and sports tourism resources in the Sichuan-Chongqing region.

Name	Region	Pearson correlation			
Destination	Sichuan	0.806	Sichuan	Sports Tourism Resources	0.885
	Chongqiong	−0.058			
Circuit	Sichuan	0.810			
	Chongqiong	−0.076	Chongqiong		0.119
Events	Sichuan	0.817			
	Chongqiong	0.231			

There exists a positive correlation between economic growth and the development of tourism (37). A region's higher GDP level, as an indication of economic prosperity, provides a solid financial foundation and material resources for the tourism industry. This economic strength not only directly promotes the construction and maintenance of tourism infrastructure (38), such as transportation networks, accommodation facilities, and tourism information services (39), but also provides necessary financial support for the marketing, product development, and human resource training of the tourism industry through taxation and government budget allocation. Moreover, regions with high GDP are usually accompanied by higher income levels among residents, which not only increases the local population's tourism consumption capacity but also provides a stable domestic market demand for the tourism industry. At the same time, economic prosperity also promotes technological progress and innovation, bringing new service models and experience methods to the tourism industry, such as the application of smart tourism and virtual reality technology. These innovations further enhance the attractiveness and competitiveness of tourist destinations.

The GDP growth in Sichuan Province has provided significant financial support and material guarantees for the development of the sports tourism industry. The Sichuan Provincial Government has actively promoted the prosperity of sports tourism by implementing a series of strategic policies and investment plans. For example, the Sichuan Provincial Government has increased investment in sports infrastructure, including the construction of sports venues, outdoor sports bases, and hiking trails, to meet the growing demand for sports tourism (40). In addition, Sichuan Province has enhanced its international visibility by hosting international sports events, such as marathons and the Sichuan International Cycling Race (41), attracting a large number of domestic and foreign tourists. The government also encourages and supports local enterprises to participate in the development of sports tourism products, promoting the diversified development of the sports tourism industry by providing financial incentives and tax benefits. The Sichuan Provincial Government also focuses on the integration of sports tourism and local culture, enhancing the cultural content and attractiveness of sports tourism by promoting sports activities with local characteristics, such as martial arts and dragon boat races. As the capital of Sichuan Province, Chengdu's GDP is far ahead of other regions in the province, and therefore, sports tourism resources are mainly concentrated here, especially sports events. This not only showcases the image of Sichuan Province but also stimulates consumption, making an important contribution to GDP growth.

In contrast to Sichuan Province, Chongqing's data and image analysis reveal a completely different situation, which is related to Chongqing's terrain, administrative area, and the characteristics of sports tourism itself. Firstly, Chongqing is characterized by its mountainous terrain. Apart from the nine main urban districts such as Yuzhong District, Dadukou District, Jiangbei District, Shapingba District, Jiulongpo District, Nan'an District, Beibei District, Yubei District, and Banan District, the GDP and tourism development in other areas are relatively slow (42). Chongqing's sports tourism resources mostly rely on mountain and water projects. For example, in the sports tourism demonstration bases, there is the Changshou Lake Sports Tourism Demonstration Base, in the sports tourism routes, there is the Ba Nan Humanistic Landscape Sports Leisure Route, and in the sports events, there is the Fu River Dragon Boat Race. These projects are all located outside the main urban areas. It can be concluded that the slow GDP development in areas outside the main urban district and the distribution of mountain and water projects in less developed areas are the reasons for the lack of correlation between GDP and the distribution of resources. Secondly, although Chongqing is a directly-administered municipality, its administrative area and economic volume are relatively small compared to Sichuan Province, which is also a reason for the lack of correlation between GDP and the distribution of resources.

#### 3.4.3 The impact of terrain on sports tourism resources in the Sichuan-Chongqing region

**Figure 8:**
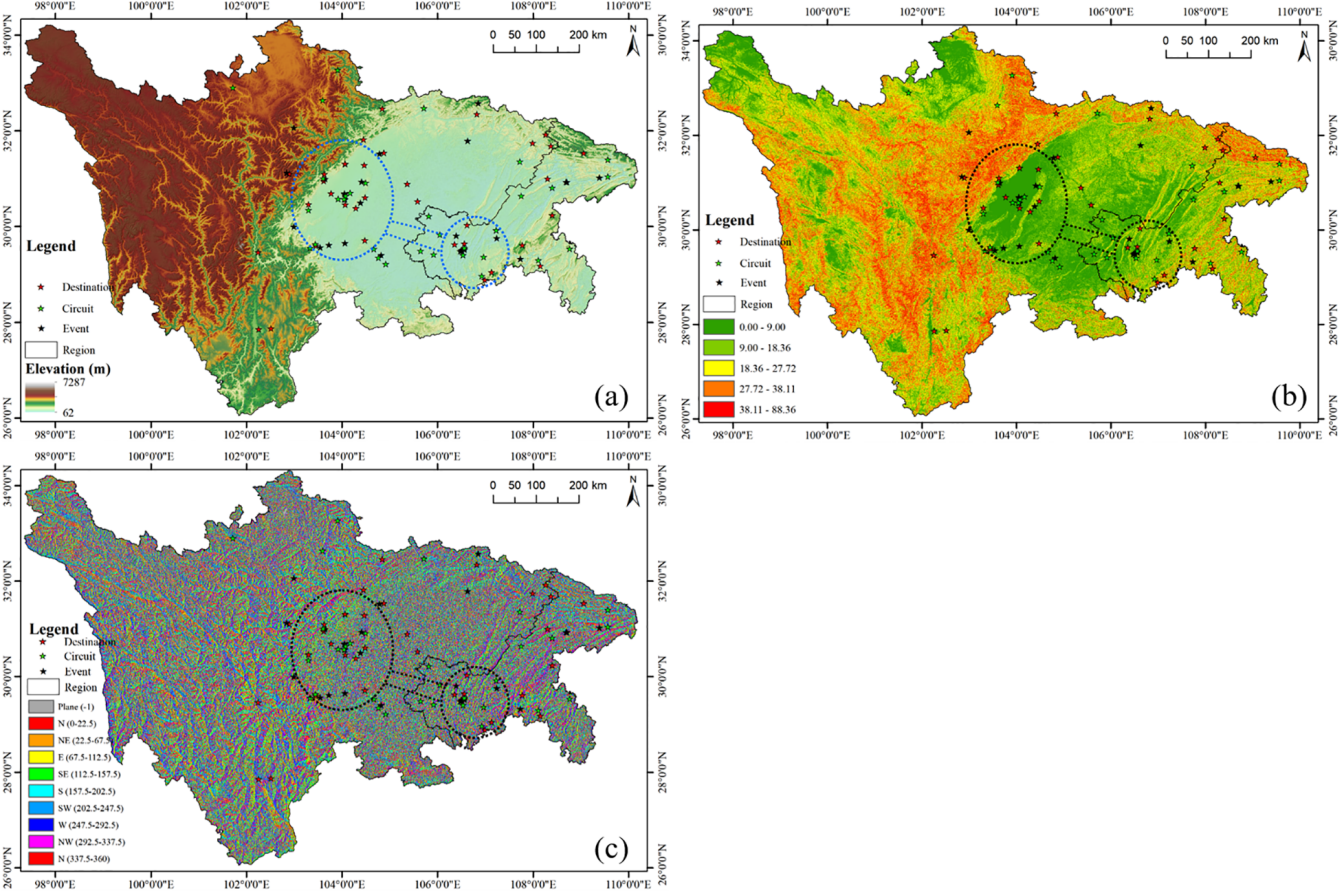
Topography effect on sports tourism resources in Sichuan and Chongqing regions. **(a–c)** are the elevation, slope, slope aspect effect on sport tourism resources, respectively.

Elevation is a significant factor affecting tourism resources (43), as natural factors such as sunlight, temperature, and soil vary across different elevations, thereby exerting a certain influence on the distribution of tourism resources. As shown in Figure 8, the vast majority of sports tourism resources in Sichuan Province are inclined to be distributed in plain areas, with only a small number in mountainous regions, attributable to the fact that these tourism resources are categorized as mountain-based projects. The sports tourism resources in Chongqing are mostly distributed in river plains and mountainous areas, where the terrain is moderate and relatively flat with good water resources, making them excellent sites for sports tourism resources. The elevation data were extracted and statistically grouped into six categories, and the heights at which sports tourism bases, routes, and events are located were tallied. As shown in Table 3, the majority of sports tourism bases in the Sichuan-Chongqing region are distributed at 0–600 m with 17 locations, followed by 601–1,000 m with 9 locations, and the remaining elevations host 3–2 locations; tourism routes in the region are predominantly concentrated between 171 and 698 m with 24 locations, followed by 699–1,226 m with 4 locations, and the remaining elevations have 0–1 location; sports events in the region are largely concentrated at 0–838 m with 29 locations, and the remaining have 0–1 location.

**Table 3:** The sports tourism resources in the Sichuan-Chongqing area have a range of altitudes.

Elevation	Destination	Elevation	Circuit	Elevation	Events
0–600 m	17	171–698 m	24	0–838 m	29
601–1,000 m	9	699–1,226 m	4	839–1,676 m	1
1,001–1,400 m	3	1,227–1,754 m	0	1,677–2,514 m	1
1,401–1,800 m	3	1,755–2,282 m	1	2,515–3,352 m	0
1,801–2,200 m	2	2,283–2,810 m	1	3,353–4,190 m	0
2,201–5,653 m	2	2,811–3,277 m	1	4,191–4,376 m	1

#### 3.4.4 Slope as an influencing factor on sports tourism resources in the Sichuan-Chongqing region

**Figure 9:**
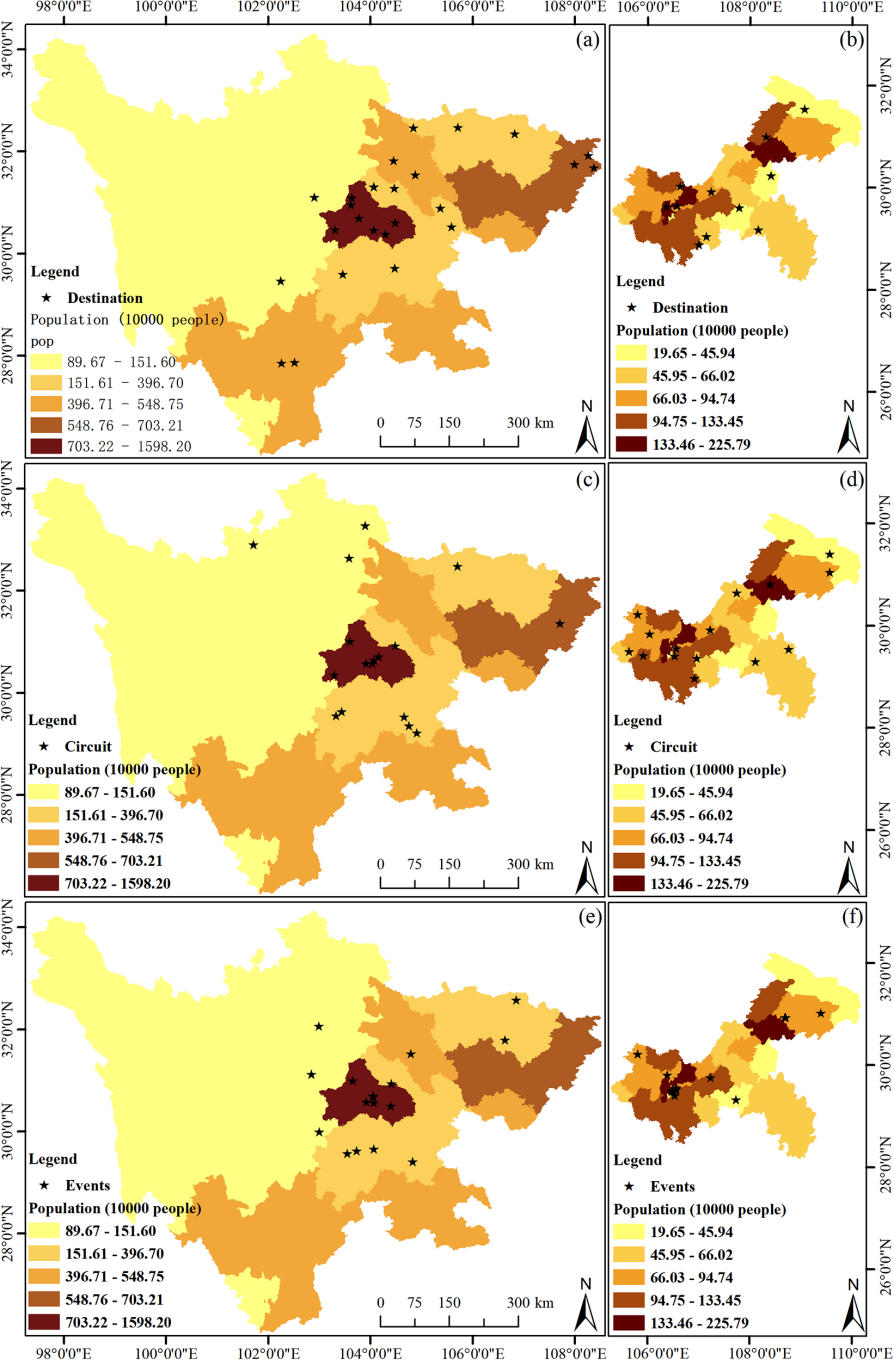
Population effect on sports tourism resources in Sichuan and Chongqing. **(a)** the population effect on the destination in Sichuan, **(b)** the population effect on the destination in Chongqing, **(c)** the population effect on the circuit in Sichuan, **(d)** the population effect on the circuit in Chongqing, **(e)** the population effect on the events in Sichuan, **(f)** the population effect on the events in Chongqing.

Slope gradient is an important indicator that reflects the macroscopic undulation of terrain (44). Sports tourism resources, due to the nature of different projects, have varying requirements for geographical resources. As shown in Figure 9, the central and eastern parts of Sichuan Province have a gentler slope, while the western region has a higher slope with a limited number of sports tourism routes that align with the topographical conditions. As indicated in Table 4, after statistical analysis of the slope gradients at which sports tourism resources are located in the Sichuan-Chongqing region, it is found that the vast majority of these resources are distributed within a gradient of 0.00–9.00°. Among the sports tourism demonstration bases, six are found within the range of 9.00–18.36°; for sports tourism routes, five are distributed within the same gradient; and two events are situated within the 9.00–18.36° range. It can be concluded that the 9.00–18.36° gradient range is the second most common distribution area for all types of sports tourism resources.

**Table 4:** Analysis of slope gradient in the distribution of sports tourism resources in the Sichuan-Chongqing region.

Slope Gradient	Destination	Circuit	Events
0.00–9.00°	21	23	29
9.00–18.36°	6	5	2
18.36–27.72°	4	3	0
27.72–38.11°	4	1	1
38.11–88.36°	2	0	0

In the realm of sports tourism, the influence of topographical features, especially slope gradients, on the distribution of resources is a multi-dimensional phenomenon. It encompasses theories and practices from various disciplines including geography, exercise physiology, psychology, and sociology. Sichuan Province and Chongqing Municipality, with their complex and varied geographical characteristics, provide abundant natural conditions for sports tourism. As a significant attribute of terrain, slope gradient directly affects the feasibility, safety, and quality of experience in sports tourism activities. From a geographical perspective, slope gradient determines the undulation of the terrain, thereby influencing the type and layout of sports activities. A gradient of 0°–9.00° provides suitable geographical conditions for a variety of sports activities. For instance, flat terrain is ideal for endurance sports such as marathons (45) and cycling races, while a moderate gradient is suitable for activities requiring physical strength and skill, such as cross-country running and mountain biking. This diversity in terrain not only meets the needs of different participants but also offers a broad range of options for sports tourism. Exercise physiology further explains the impact of appropriate gradients on participants’ physiological responses. A moderate gradient can increase the difficulty of exercise, thereby enhancing participants’ cardiorespiratory function and muscle strength (46). However, an excessively steep gradient may lead to excessive intensity and an increased risk of injury (47). Yet, some sports naturally occur in areas with steeper gradients, such as skiing or mountain biking. From a psychological standpoint, the focus is on the participants’ internal experiences. An appropriate gradient can stimulate the interest of athletes, with a gradient of 0.00°–9.00° offering a moderate level of challenge that meets participants’ psychological needs without causing excessive psychological pressure, thereby enhancing the appeal of sports tourism.

In summary, an appropriate gradient not only provides the physical conditions for sports activities but also enhances the tourism experience by offering beautiful natural landscapes and rich ecological experiences. Moreover, the development of sports tourism can drive local economic growth, promote community participation and cultural heritage, achieving sustainable development in social, economic, and environmental terms (48).

Slope aspect, as a significant dimension of topography, exerts a profound influence on the conduct of sports tourism activities (49). Sunny slopes, due to their advantageous sunlight conditions, have become hotspots for the distribution of sports tourism resources. Firstly, from a geographical perspective (Table 5), slope aspect has a notable impact on climatic conditions. Sunny slopes, receiving more solar radiation, have relatively higher temperatures (50), providing suitable climatic conditions for sports tourism activities, while some sports tourism resources may require the shade of the north slopes. For instance, ski resorts on sunny slopes may have unstable snow quality due to higher temperatures, making them unsuitable for skiing (51). Moreover, vegetation on sunny slopes is lusher (52), offering a favorable natural environment for outdoor sports such as mountain biking and hiking. Secondly, from the perspective of exercise physiology, the warm climate of sunny slopes helps improve athletes’ thermoregulation efficiency and reduces the risk of sports injuries, providing favorable conditions for hosting sports events. Research indicates that exercising in a warm environment result in relatively lower cardiorespiratory demands on athletes, allowing for better athletic performance (53). Furthermore, from a psychological standpoint, the sunlight conditions on sunny slopes can enhance the mood states of athletes and tourists. Sunlight promotes the synthesis of serotonin in the human body, which helps alleviate depressive moods and increase feelings of well-being (54). In addition, the natural scenery on sunny slopes is more likely to provide aesthetic pleasure, enhancing the appeal of sports tourism. Lastly, from a sociological perspective, sunny slopes are often associated with more human activities. In Sichuan and Chongqing, many sunny slope areas are traditional living areas for local residents, rich in cultural background and historical relics. The development of sports tourism activities in these areas not only stimulates local economic growth but also promotes cultural exchange and heritage.

**Table 5:** Analysis of slope aspect in the distribution of sports tourism resources in the Sichuan-Chongqing region.

Slope Aspect	Destination	Circuit	Events
N (0–22.5)	1	3	1
NE (22.5–67.5)	4	3	6
E (67.5–112.5)	5	2	5
SE (112.5–157.5)	5	2	4
S (157.5–202.5)	8	3	6
SW (202.5–247.5)	7	6	4
W (247.5–292.5)	4	6	4
NW (292.5–337.5)	3	4	2
N (337.5–360)	0	3	0

In summary, the sunny slope terrain in Sichuan and Chongqing provides unique conditions for the distribution of sports tourism resources. The sunlight advantage, warm climate, rich vegetation, and profound cultural background of sunny slopes collectively promote the concentrated distribution of sports tourism activities. These factors not only meet the basic needs of sports tourism activities but also enhance the quality of participants’ experiences and promote the sustainable development of the sports tourism industry.

#### 3.4.5 The impact of population density on sports tourism resources

Population density, as a core parameter measuring the intensity of regional population distribution, exerts a profound and significant influence on the layout of tourism resources (55). In areas with high population density, the economies of scale and diversification characteristics of sports tourism demand are pronounced, driving a trend of intensive resource allocation and promoting the diversification and refinement of sports tourism products and services. Such areas, with a large potential base of participants and spectators, provide a solid market foundation for sports events and related activities, accelerating market response sensitivity and stimulating profound changes in the optimization and innovative development of sports tourism resources.

As shown in Figure 9, there is a positive correlation between population density and the distribution of sports tourism demonstration bases, routes, and events in Sichuan Province and Chongqing, for example, Chengdu, the city with the highest population density in Sichuan Province, has a higher distribution of sports resources and shows a trend of being a single-tier city.

Furthermore, areas with high population density are often accompanied by highly developed infrastructure such as transportation networks and communication facilities (56). The completeness of these infrastructures greatly enhances the accessibility and convenience of sports tourism activities, providing visitors with a smoother and more efficient travel experience. In addition, these infrastructures also act as “catalysts” for the development of the sports tourism industry, promoting further expansion and deepening of the sports tourism market by enhancing regional connectivity and information efficiency.

From the perspective of economic effects, the distribution of sports tourism resources in areas with high population density demonstrates multiple positive spillover effects. The hosting of sports events and activities not only directly promotes rapid local economic growth but also significantly increases employment opportunities and tax revenue by driving the development of related industry chains. At the same time, these activities also serve as effective carriers for enhancing the brand image and international visibility of cities, strengthening the city's soft power and comprehensive competitiveness through media exposure and word-of-mouth, injecting new vitality and momentum into the city's long-term development.

In summary, factors such as transportation accessibility, regional Gross Domestic Product (GDP) levels, natural geographical characteristics like elevation and slope, the complexity of terrain aspect, and the distribution of population density have significantly influenced the distribution pattern and dynamic evolution of sports tourism resources in this area. However, the primary factors governing the spatiotemporal distribution of sports tourism resources in this region still require further in-depth discussion.

### 3.5 Analysis of the main driving factors for the spatiotemporal distribution of sports tourism in the Sichuan-Chongqing region

To further dissect the causes of the spatiotemporal distribution of sports tourism resources in this area, a GeoDetector was utilized for a quantitative analysis of factors such as transportation, GDP, elevation, and population density. The q values are calculated according to Equation 5. This analysis aims to identify the primary controlling factors of sports tourism resources in the region.

As shown in Table 6, among the four influencing factors of transportation, GDP, population, and elevation, in Sichuan Province, the factor with the greatest impact on the distribution of sports tourism bases is elevation (Q value of 0.031), the factor with the greatest impact on the distribution of sports tourism routes is transportation (Q value of 0.025), and the factor with the greatest impact on sports events is population (Q value of 0.037). In Chongqing Municipality, the factors with the greatest impact on the distribution of sports tourism bases, routes, and events are elevation (Q value for bases of 0.034), elevation (Q value for routes of 0.047), and population (Q value of 0.038), respectively.

**Table 6:** Analysis of the main driving factors of the spatiotemporal distribution of sports tourism in the Sichuan-Chongqing region.

Name	Region	Q Statistic			
		Transportation	GDP	Population	Altitude
Destination	Sichuan	0.011	0.016	0.001	**0** **.** **031**
	Chongqiong	0.004	0.019	0.014	**0**.**034**
Circuit	Sichuan	**0**.**025**	0.005	0.002	0.002
	Chongqiong	0.024	0.038	0.042	**0**.**047**
Events	Sichuan	0.028	0.015	**0**.**037**	0.026
	Chongqiong	0.001	0.006	**0**.**038**	0.007

Bolded values are the maximum in that row and are the main driving factors.

**Figure 10:**
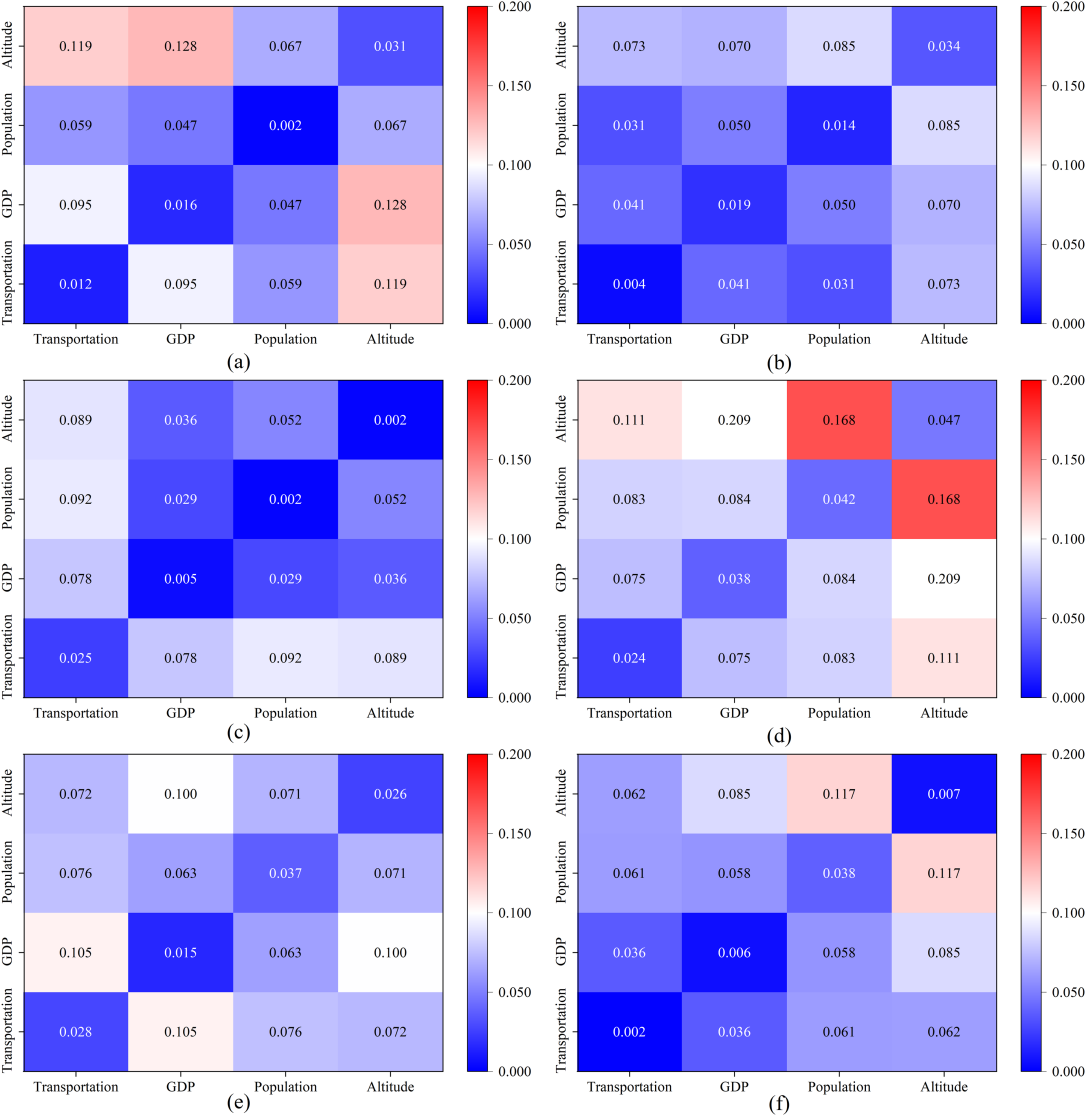
Analysis of the interactive effects of factors influencing the distribution of sports tourism resources in the Sichuan-Chongqing Region. **(a)** the driving factors of the destination in Sichuan, **(b)** the driving factors of the destination in Chongqing, **(c)** the driving factors of the circuit in Sichuan, **(d)** the driving factors of the circuit in Chongqing, **(e)** the driving factors of the events in Sichuan, **(f)** the driving factors of the events in Chongqing.

To further explore the interactive effects of various influencing factors on sports tourism resources, calculations were made using the GeoDetector, as illustrated in Figure 10. From (a), it can be observed that among the four factors affecting the distribution of sports tourism demonstration bases in Sichuan Province, the interaction between GDP and elevation is the most significant. From (c), it is evident that among the four factors influencing the distribution of sports tourism routes in Sichuan Province, there is a notable interactive effect between transportation and population. From (e), it is apparent that among the four factors impacting sports events in Sichuan Province, there is a significant interactive effect between transportation and GDP. From (b), it can be discerned that among the four factors influencing the distribution of sports tourism demonstration bases in Chongqing Municipality, there is a significant interactive effect between population and elevation. From (d), it is clear that among the four factors affecting the distribution of sports tourism routes in Chongqing Municipality, there is a significant interactive effect between GDP and elevation. From (f), which pertains to the distribution of sports events in Chongqing Municipality, there is a significant interactive effect between population and elevation.

Therefore, the dominant controlling factors for the spatiotemporal distribution of each type of sports tourism resource differ, and the degrees of interactive effects among the influencing factors are also distinct.

## 4 Conclusion

This study that analyzes the spatial-temporal characteristics of sports tourism resource distribution, providing a clear understanding of the current status, developmental trends, and influencing factors in the Sichuan-Chongqing region. To elucidate these spatial-temporal features, we employed a GIS-based spatial analysis to map the distribution of sports tourism resources in Sichuan and Chongqing. This analysis leveraged buffer zones, kernel density estimation, and equilibrium analysis to explore the spatial patterns. The research findings are as follows: (1) The temporal distribution of sports tourism resources in Sichuan-Chongqing can be primarily divided into two phases: an exploratory and ascending stage, followed by a high-quality development stage. (2) In Sichuan, sports tourism demonstration bases are predominantly located in the central and eastern regions, with no presence in the western region. These bases form the cornerstone, while routes are primarily distributed in the central and northern areas, excluding the western and southern regions from premium routes. Event venues are relatively concentrated, primarily in Chengdu. (3) In Chongqing, sports tourism bases exhibit a trend of higher concentrations in the central region and lower densities in peripheral areas, though no specific clustering is observed. The distribution of premium routes is characterized by fewer in the center and more evenly spread around the periphery, lacking centralized patterns. Events are concentrated within the main urban areas. (4) An analysis of the impact of various factors, including elevation, transportation, GDP, and population, on sports tourism resources revealed that elevation, transportation, and population density exert the most significant influences on the distribution of bases, routes, and events in Sichuan. In contrast, for Chongqing, elevation and population density are the primary determinants for both bases and events, while elevation also plays a crucial role in route distribution.

Based on the current distribution of sports tourism resources in the Sichuan-Chongqing region, the following development suggestions are proposed: (1) In response to the lack of sports tourism demonstration bases in western Sichuan, it is recommended to promote the construction of sports tourism infrastructure in this region through policy support and capital investment. By developing sports tourism projects that are compatible with local natural resources, regional balanced development can be achieved. (2) Given that Chengdu is a major hub for sports tourism activities, it should further enhance service quality and diversify sports tourism product offerings to cater to the needs of different tourists. Additionally, strengthening the branding of sports tourism and enhancing Chengdu's competitiveness in the domestic and international sports tourism markets are crucial. (3) Chongqing should leverage the high concentration of sports tourism bases in its central area to further develop sports tourism products that integrate with the city's unique characteristics. At the same time, encouraging peripheral regions to develop distinctive sports tourism projects based on local resources can promote balanced development of sports tourism across the entire municipality. (4) Considering the significant impact of altitude, transportation, and population density on the distribution of sports tourism resources, it is suggested to develop special sports tourism activities such as mountaineering and skiing in high-altitude areas. Furthermore, optimizing the transportation network to improve the accessibility of sports tourism resources and hosting large-scale sports events in densely populated areas can fully harness the market potential brought by population density.

## Data Availability

The original contributions presented in the study are included in the article/Supplementary Material, further inquiries can be directed to the corresponding author.
